# A Rare Case Report of ST-Segment Elevation Myocardial Infarction and Recurrent Chest Pain in Polyarteritis Nodosa

**DOI:** 10.7759/cureus.16157

**Published:** 2021-07-04

**Authors:** Pradeep Kumar Devarakonda, Vishal R Dhulipala, Monika Karki, Samir Garyali, Sarath Reddy

**Affiliations:** 1 Internal Medicine, The Brooklyn Hospital Center, Brooklyn, USA; 2 Cardiology, Mount Sinai Hospital, New York, USA; 3 Cardiology, The Brooklyn Hospital Center, Brooklyn, USA; 4 Cardiology, Mount Sinai Heart, Brooklyn, USA

**Keywords:** internal medicine and rheumatology, adult cardiology, st-elevation myocardial infarction (stemi), clinical case report, polyarteritis nodosa

## Abstract

Polyarteritis nodosa (PAN) is a type of vasculitis that mainly affects small and medium-sized blood vessels. The clinical presentation can be nonspecific as weight loss, abdominal pain, and hypertension, or fatal as myocardial infarction (MI) and bowel perforation depending upon the organ involved. Cardiac involvement of PAN usually manifests as congestive heart failure, aneurysms, or MIs and is mostly identified during postmortem studies of autopsied patients. Here, we report a case of anterior MI as a sequela of PAN in a 40-year-old female who was diagnosed with PAN two weeks before her MI. She presented with intermittent chest pain for one day. At the time of admission, an electrocardiogram revealed anterior MI, and she was subsequently found to have 95-99% stenosis of the proximal left anterior descending artery during cardiac catheterization. The patient was successfully treated with percutaneous coronary intervention and was started on dual antiplatelet therapy. Her treatment was continued with steroids and cyclophosphamide. The case illustrates the importance of recognizing MI as a sequela of PAN as timely treatment could be lifesaving.

## Introduction

Polyarteritis nodosa (PAN) is a rare condition with an estimated annual incidence of three to five cases per 1,000,000 cases. However, the incidence may be slightly higher in hepatitis B endemic places [1]. PAN was first described in 1866 by Kussmaul and Maier as a focal, inflammatory, arterial nodule and was termed as “periarteritis nodosa,” which was later changed to “polyarteritis nodosa” as inflammation involved the entire arterial wall. Cardiac involvement is commonly seen in PAN, and antineutrophil cytoplasmic antibodies (ANCA) are usually negative in PAN. The pathophysiology of cardiac involvement is caused by inflammation mechanism, and coronary artery involvement is usually described as a destructive and productive type of vasculitis [2].

## Case presentation

**Figure 1:**
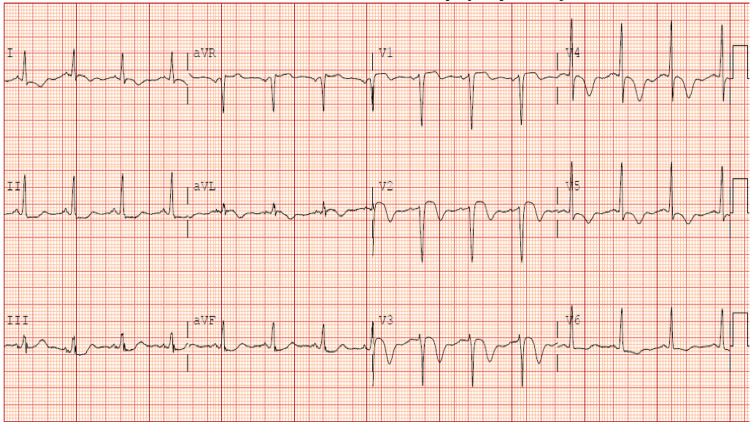
ECG on presentation showing sinus rhythm with T-wave inversions in precordial and lateral leads and ST-segment elevation in the precordial leads. ECG: electrocardiogram

A 40-year-old female with a past medical history of hypertension, asthma, intracranial aneurysm post-craniotomy with clipping, and recently diagnosed PAN, deep venous thrombosis, celiac artery occlusion, and superior mesenteric artery stenosis presented with chest pain. She described the pain to be pressure-like, intermittent, lasting for 15 minutes, and associated with nausea and vomiting. On physical examination, the patient was afebrile, had a blood pressure of 166/118 mmHg, a heart rate of 92 beats per minute, and SpO_2_ of 100% on room air. The patient was noted to have ecchymosis on the right thigh, but normal physical examination findings. An electrocardiogram (ECG) on admission demonstrated sinus rhythm with T-wave inversions in precordial and lateral leads and ST-segment elevation in the precordial leads (Figure 1). Labs revealed elevated serum cardiac enzyme troponin I to 1.56 ng/mL (normal: 0-0.3 ng/mL) sedimentation rate of 5 mm/hour (normal: 0-15 mm/hour), a normal diabetic profile, and normal C-reactive protein of 0.78 mg/L (normal: 0-10 mg/L). Before the current admission, the patient’s autoimmune workup showed a positive antinuclear antibody screen and positive lupus anticoagulant but was negative for ANCA, anti-dsDNA, and hepatitis B surface antigen. She was recently diagnosed with PAN based on weight loss of nearly 4.5 kg in six months, diastolic blood pressure of 114 mmHg, intracranial aneurysm, and involvement of a medium vessel (superior mesenteric artery) with an area of beading seen on CT abdomen with contrast and angiogram.

**Figure 2:**
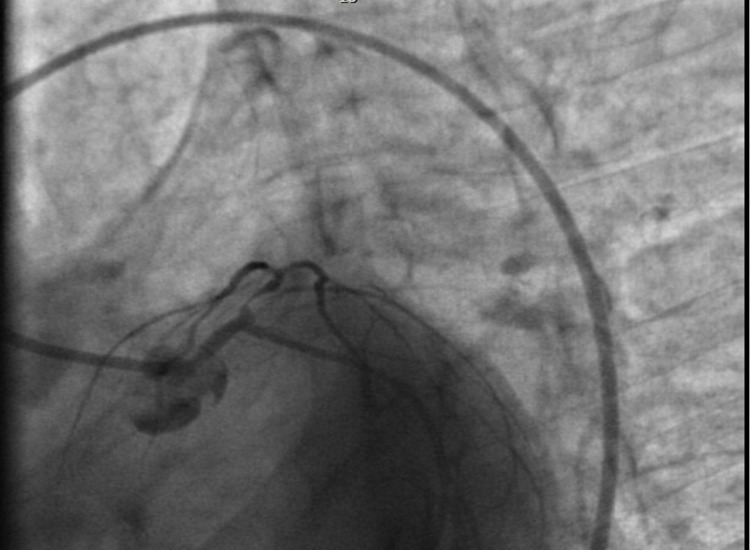
Left coronary system with a 6-F guiding catheter and left anterior oblique. Caudal view before PCI. PCI: percutaneous coronary intervention

**Figure 3:**
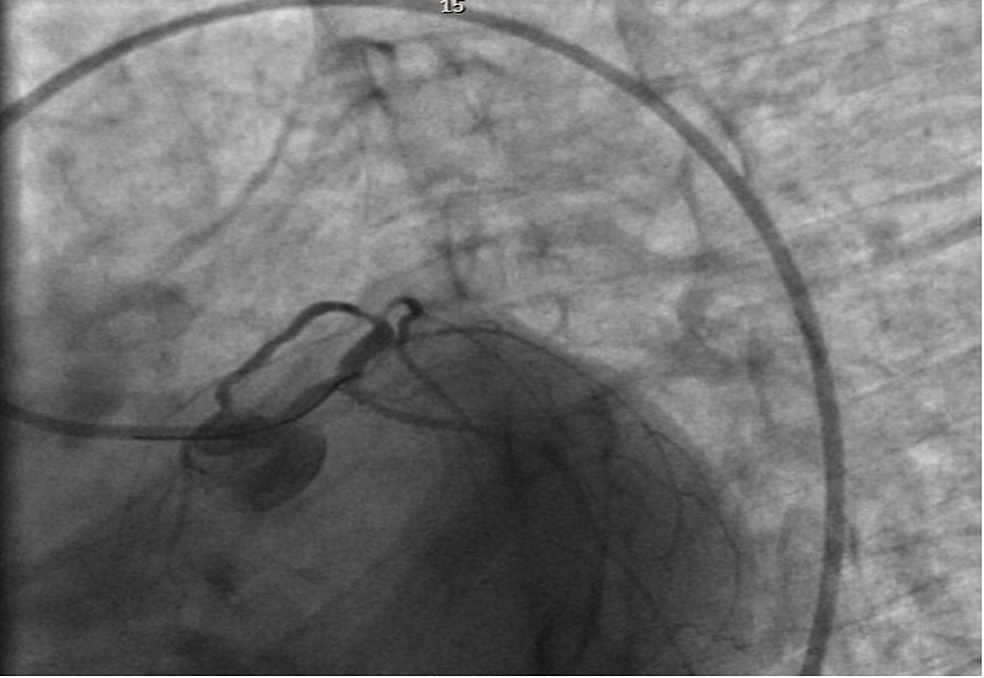
Left coronary system with a 6-F guiding catheter and left anterior oblique. Caudal view after PCI. PCI: percutaneous coronary intervention

During the current admission, the patient was taken to the cardiac catheterization lab directly from the emergency department due to ECG findings and positive troponin. Left heart catheterization (LHC) revealed the dominant coronary system with small caliber coronary arteries: normal left main artery, 95-99% focal thrombotic lesion of proximal left anterior descending (LAD) artery, moderately diffuse diseases ramus intermedius, and luminal irregularities of the left circumflex artery and right coronary artery (Figure 2). The patient underwent percutaneous coronary intervention with a 1.5 mm × 12 mm balloon to 8 atm for eight seconds of inflation, and a 3.0 mm × 12 mm bare-metal stent (BMS) at 14 atm for eight seconds of inflation in the proximal LAD (Figure 3). She was continued on dual antiplatelet therapies. Transthoracic echocardiography revealed a left ventricular ejection fraction of 50-55%, mild tricuspid regurgitation, but no aneurysms. The patient was later readmitted several times for chest pain within a few days after LHC despite being on antianginal medications such as Imdur and beta-blockers. During one of the admissions, her troponin was elevated up to 8 ng/mL and ECG revealed ST elevations in II, III, and aVF. Repeat LHC showed a patent proximal LAD with a BMS. Subsequently, during another admission for angina, ECG revealed T-wave inversions in lateral leads and elevated troponin (0.54 ng/mL), which resolved later and was likely secondary to vasospasm. There are very few studies regarding the specific treatment of acute coronary syndrome in patients with PAN. In this case, we continued the standard medical treatment with steroids and cyclophosphamide for PAN management and used dual antiplatelet therapy post-BMS and continued use of antianginal medications.

## Discussion

PAN is a type of vasculitis that involves necrotizing inflammation of the small and medium-sized blood vessels [3]. It occurs in about 3 to 4.5 cases per 100,000 people yearly in the United States with an average age of onset of 40 with a male predominance [1]. A clinicopathology meeting in 1948 published the first report on coronary artery involvement in adult patients with PAN [4]. The etiology of PAN can be idiopathic or can be in the setting of infections such as hepatitis B virus or several other organisms such as *Pseudomonas*, *Klebsiella*, parvovirus B-19, *Toxoplasma*, *Trichinella*, and *Yersinia *species. PAN is also associated with conditions such as hairy cell leukemia, Sjogren’s syndrome, and rheumatoid arthritis involving one or multiple organs except for the lungs. Diagnosis of PAN relies on clinical, angiography, and biopsy findings. The diagnosis of PAN requires the presence of three or more of the 10 criteria published by the American College of Rheumatology: weight loss of ≥4 kg, livedo reticularis, testicular pain or tenderness, myalgias, mono- or polyneuropathy, diastolic blood pressure of >90 mmHg, elevated blood urea nitrogen or serum creatinine levels, presence of hepatitis B reactants in the serum, arteriographic abnormality, presence of granulocyte, or mixed leukocyte infiltrate in an arterial wall biopsy [5]. Cardiac involvement is reported in 35% of cases with PAN, and it usually manifests as congestive heart failure, coronary aneurysms, or myocardial infarction likely due to coronary arteritis, renal involvement, and hypertension [6]. Most often cardiac involvement is silent and nonspecific, which makes it difficult to diagnose MI-related PAN [7,8]. Management of PAN with cardiac involvement is still challenging as there is very little consensus on optimal therapy. Coronary artery lesions are usually managed with percutaneous coronary intervention and coronary artery bypass graft (CABG). The first coronary angiogram in an adult patient with PAN was published by Przybojewski et al., demonstrating its safety in PAN patients [9]. PAN with a high five-factor score of >2 (age >65, proteinuria, renal insufficiency, severe gastrointestinal involvement, central nervous system involvement, and cardiomyopathy) have a better prognosis with cyclophosphamide along with prednisone [10]. Patients with PAN have a survival rate of 13% in five years without any treatment but it increases up to 80% with appropriate treatment [1].

Our patient was categorized as having moderate-to-severe PAN as there was evidence of cardiac and gastrointestinal involvement. She was treated with both glucocorticoids and cyclophosphamide, along with a BMS to LAD, and was started on antiplatelet and anticoagulation with warfarin (due to deep venous thrombosis). The BMS was selected in this patient because of recently diagnosed gastric ulcers, which makes patients more prone to bleed on warfarin and dual antiplatelet therapy for a longer duration.

## Conclusions

PAN should be considered in young patients who are recently diagnosed with hypertension, aneurysms, or coronary lesions of unknown causes. Hepatitis, other causes of infections, and extensive autoimmune workup are required to know the etiology. Although the primary therapy is immunosuppression, cardiac medications such as beta-blockers, diuretics, antithrombotics, angiotensin-converting enzyme inhibitors, and antiarrhythmic medicines, pacemaker implantation may be needed for the management of a few patients. Proper management of PAN with cardiac involvement is still challenging as it requires further investigation and more studies. With optimal medical management, the prognosis of these patients can be increased and timely treatment can be lifesaving.

## References

[1] Jese R, Rotar Z, Praprotnik S, Hocevar A, Tomsic M (2014). AB0581 The estimated annual incidence rate of polyarteritis nodosa in Slovenia. Ann Rheum Dis.

[2] O Zimba, M Bagriy (2017). Cardiac involvement in polyarteritis nodosa: a retrospective pathological study of 37 autopsy cases. Ann Rheum Dis.

[3] Colmegna I, Maldonado-Cocco JA (2005). Polyarteritis nodosa revisited. Curr Rheumatol Rep.

[4] Lai J, Zhao L, Zhong H (2021). Characteristics and outcomes of coronary artery involvement in polyarteritis nodosa. Can J Cardiol.

[5] Garg R, Joe D, Abbott JD (2020). A case of acute thrombotic myocardial infarction in polyarteritis nodosa. R I Med J (2013).

[6] McWilliams ET, Khonizy W, Jameel A (2013). Polyarteritis nodosa presenting as acute myocardial infarction in a young man: importance of invasive angiography. Heart.

[7] Odhav S, McKown K, Lohr KM (1994). Polyarteritis nodosa presenting as recurrent myocardial infarction. Chest.

[8] Gayraud M, Guillevin L, le Toumelin P, Cohen P, Lhote F, Casassus P, Jarrousse B (2001). Long-term followup of polyarteritis nodosa, microscopic polyangiitis, and Churg-Strauss syndrome: analysis of four prospective trials including 278 patients. Arthritis Rheum.

[9] Przybojewski JZ (1981). Polyarteritis nodosa in the adult. Report of a case with repeated myocardial infarction and a review of cardiac involvement. S Afr Med J.

[10] Schrader ML, Hochman JS, Bulkley BH (1985). The heart in polyarteritis nodosa: a clinicopathologic study. Am Heart J.

